# The MCP2 and the wrist plus two extensor compartments are the most affected and responsive joints/tendons out of the US7 score in patients with rheumatoid arthritis-an observational study

**DOI:** 10.1186/s13075-022-02874-y

**Published:** 2022-08-05

**Authors:** A. F. Podewski, A. M. Glimm, I. Fischer, G. A. W. Bruyn, P. Hanova, H. B. Hammer, A. B. Aga, E. A. Haavardsholm, S. Ramiro, G. R. Burmester, M. Backhaus, S. Ohrndorf

**Affiliations:** 1 Department of Rheumatology and Clinical Immunology, Charité – Universitätsmedizin Berlin, Berlin, Germany; 2 Department of Internal Medicine - Rheumatology and Clinical Immunology, Park-Klinik Weißensee, Berlin, Germany; 3 Department of Endocrinology, Nephrology, Rheumatology, Division Rheumatology, Universitätsklinikum Leipzig, Leipzig, Germany; 4 Biostatistics Tubingen, Tubingen, Germany; 5 Department of Rheumatology, MC Groep Hospitals, Lelystad, Netherlands; 6 Department of Rheumatology, First Faculty of Medicine, Charles University of Prague, Prague, Czech Republic; 7 Center for treatment of Rheumatic and Musculoskeletal Diseases (REMEDY), Diakonhjemmet Hospital, Oslo, Norway; 8 Faculty of Medicine, University of Oslo, Oslo, Norway; 9 Department of Rheumatology, Leiden University Medical Center, Leiden, The Netherlands; 10 Zuyderland Medical Center, Heerlen, The Netherlands

**Keywords:** Ultrasonography, Arthritis, Rheumatoid, Synovitis

## Abstract

**Background:**

There is no international consensus on an optimal ultrasound score for monitoring of rheumatoid arthritis (RA) on patient-level yet. Our aim was to reassess the US7 score for the identification of the most frequently pathologic and responsive joint/tendon regions, to optimize it and contribute to an international consensus. Furthermore, we aimed to evaluate the impact of disease duration on the performance of the score.

**Methods:**

RA patients were assessed at baseline and after 3 and 6 months of starting/changing DMARD therapy by the US7 score in greyscale (GS) and power Doppler (PD). The frequency of pathologic joint/tendon regions and their responsiveness to therapy were analyzed by Friedman test and Cochrane-Q test respectively, including the comparison of palmar vs. dorsal regions (chi-square test). The responsiveness of different reduced scores and the amount of information retained from the original US7 score were assessed by standardized response means (SRM)/linear regression. Analyses were also performed separately for early and established RA.

**Results:**

A total of 435 patients (*N* = 138 early RA) were included (56.5 (SD 13.1) years old, 8.2 (9.1) years disease duration, 80% female). The dorsal wrist, palmar MCP2, extensor digitorum communis (EDC) and carpi ulnaris (ECU) tendons were most frequently affected by GS/PD synovitis/tenosynovitis (wrist: 45%/43%; MCP2: 35%/28%; EDC: 30%/11% and ECU: 25%/11%) and significantly changed within 6 months of therapy (all *p* ≤0.003 by GS/PD). The dorsal vs. palmar side of the wrist by GS/PD (*p* < 0.001) and the palmar side of the finger joints by PD (*p* < 0.001) were more frequently pathologic. The reduced US7 score (GS/PD: palmar MCP2, dorsal wrist, EDC and ECU, only PD: dorsal MCP2) showed therapy response (SRM 0.433) after 6 months and retained 76% of the full US7 score’s information.

No major differences between the groups of early and established RA could be detected.

**Conclusions:**

The wrist, MCP2, EDC, and ECU tendons were most frequently pathologic and responsive to therapy in both early and established RA and should therefore be included in a comprehensive score for monitoring RA patients on patient-level.

**Supplementary Information:**

The online version contains supplementary material available at 10.1186/s13075-022-02874-y.

## Background

Recent advances in the treatment for rheumatoid arthritis (RA) like conventional synthetic (cs), biological (b) and targeted synthetic (ts) disease-modifying antirheumatic drugs (DMARDs), along with treating early and to target have significantly improved patients’ outcome [1–3].

Clinical and laboratory parameters as well as sensitive and reliable imaging modalities are utilized to ensure an early diagnosis and a rapid treatment initiation to prevent joint damage.

Musculoskeletal ultrasound (MSUS) has proven to be a valid imaging method for the detection of inflammation (synovitis, tenosynovitis) and bony damage such as erosions with comparable sensitivity and specificity to magnetic resonance imaging (MRI) [4–8]. Furthermore, MSUS and MRI are more sensitive than clinical examination in detecting joint inflammation [9]. MSUS is therefore increasingly used in clinical practice and research.

Standardization of MSUS scanning techniques and definitions of pathologies are driven forward by the Outcome Measures in Rheumatology (OMERACT) ultrasound group [10–13] and the European Alliance of associations for rheumatology (EULAR) recommendations and definitions [14–17] including ultrasound synovitis and tenosynovitis scores on joint and tendon level. Furthermore, several US scoring systems, including reduced joint scores, have been developed to measure disease activity and therapeutic response [18–26], but they differ regarding the (number of) included joints and/or pathologic manifestations. The German US7 score by Backhaus et al. [19] includes the wrist, metacarpophalangeal joint (MCP) 2 and 3, proximal interphalangeal joint (PIP) 2 and 3, metatarsophalangeal joint (MTP) 2 and 5 as well as the extensor digitorum communis (EDC), extensor carpi ulnaris (ECU) and flexor tendons (superficialis/profundus) of the wrist (FTS/P), finger flexor tendons 2, 3 (FT2, FT3), and finger extensor tendons 2, 3 on MCP level (ET2, ET3) of the clinically most affected side by greyscale (GS) and power Doppler (PD) ultrasound. It examines soft tissue lesions (synovitis and tenosynovitis) and erosions. Previous studies have shown that the US7 score is feasible, reliable and sensitive to change over a 12-month-period [19, 27, 28]. The score takes 10-15 min to perform in daily practice.

Aga et al. proposed the USRA9 score [21] which showed good responsiveness, retained most information of their original full score [29] and performed better than previous scores [19, 20, 30]. It includes MCP1-3, PIP2,3, wrist (radiocarpal joint), extensor carpi ulnaris (ECU) tendon and MTP2,3. However, it only examines the dorsal aspect of the joints/tendons [21] and its feasibility in daily practice is compromised due to the long time it takes to perform.

As no consensus on an optimal ultrasound scoring system to monitor patients with RA has been internationally achieved so far, we wanted to contribute with additional information on the US7 score by reassessing it. The primary objective of the present study was to assess the existing US7 score to identify the joints and tendons, as well as the side in which they are examined (palmar/dorsal) that are most frequently pathologic and responsive during 3 and 6 months of therapy and to further investigate whether a reduced version of the US7 score, which would improve its feasibility, would still be responsive. Our secondary objective was to evaluate the impact of disease duration (early and established RA) on the performance of the score.

## Patients and methods

### Study population

Patients were recruited in 54 centers participating in the German nationwide “Sono Remission Plus” project between 2006 and 2010 [19, 27]-a prospective, observational study on patients with RA classified according to the American College of Rheumatology (ACR) criteria of 1987 [31]. The study was approved by the ethical committee of the University of Tuebingen, Germany (199/2007BO2), and all patients signed an informed consent upon inclusion. Patients were included in the study if they were starting or changing DMARD treatment. The decision for treatment start/change was taken by the treating rheumatologists according to current treatment recommendations. Therapies included first-line csDMARD therapy after new initiation, therapy switch from csDMARD to a second csDMARD, first-line biologic after csDMARD therapy and therapy switch from biologic to a second biologic. In the analysis of the “Sono Remission Plus” project, patients were divided into subgroups according to therapy [27]. The focus of the present study was on the detailed US results for the identification of the most frequently pathologic and responsive joint/tendon regions.

For the present analysis, we included patients with available data at baseline and after 3 and 6 months. Patients with missing data at baseline, 3 months, or 6 months visit were excluded.

Ultrasound was performed by rheumatologic specialists. The training of a rheumatologic specialist in Germany includes at least 300 musculoskeletal ultrasound examinations. Further information can be found in previous publications [19, 27].

### US7 score examination

In each patient, the clinically most affected hand and forefoot by tenderness and/or swelling were chosen for the US7 score examination.

The US7 score examination included the assessment of the following pathologic manifestations according to EULAR criteria [31] and OMERACT definitions [10] for greyscale (GS) and power Doppler (PD) ultrasound:

- *Synovitis* for joints: wrist (dorsal, palmar and ulnar side) each in GS/PD, metacarpophalangeal joints 2 and 3 (palmar MCP2 and palmar MCP3 in GS, palmar/dorsal in PD), proximal interphalangeal joints 2 and 3 (palmar PIP2 and palmar PIP3 in GS, palmar/dorsal in PD), and metatarsophalangeal joints (dorsal MTP2 and dorsal MTP5 in GS/PD), scored 0-3 for GS and PD separately, summed up to a total GS synovitis subscore (range 0-27) and to a total PD synovitis subscore (range 0-39) [19]
  - ◦ Examination of the wrist: In the dorsal aspect, the probe was parallel to the extensor digitorum tendons (dorso-median). For the palmar wrist examination, the probe was placed parallel to the median nerve (palmomedian), and for the ulnar aspect, the probe was set parallel to the extensor carpi ulnaris tendon [19]
- *Tenosynovitis/paratenonitis* for tendons: extensor compartment IV = extensor digitorum communis (EDC), VI = extensor carpi ulnaris (ECU) and flexor tendon (superficialis/ profundus) of the wrist (FTS/P), finger flexor tendons 2, 3 (FT2, FT3), and finger extensor tendons 2, 3 on MCP level (ET2, ET3), scored in grades 0/1 for GS and grades 0-3 for PD, summed up to a total GS tenosynovitis subscore (range 0-7) and to a total PD tenosynovitis subscore (range 0-21) [19]
- Erosions were not included in the present analysis due to missing data

Musculoskeletal ultrasound was performed using a 10-18 MHz linear scanner and middle class to high-end machine US devices. Settings for GS were defined by a frequency of > 10 MHz, the use of GS gain depending on the joint regions and patients was on average 50%. Settings for PD were set as follows: frequency: 9.1 MHz, pulse repetition frequency: 500-750 Hz (depending on machine setting), PD gain depended on joint regions and patients and was average 50%, and wall filter was low for example, 3, and had to be maintained throughout the study. The PD gain was not supposed to change within a joint panel of a patient during the examination. The exact same machine had to be used on every patient during the study time (compare [27]).

PD scoring of synovial/tenosynovial vascularity was performed semi-quantitatively (grades 0-3) according to Szkudlarek et al. [4]. GS synovitis (effusion and synovial hypertrophy combined) was scored semi-quantitatively (0-3) as described by Scheel et al. [32]. Tenosynovitis/paratenonitis in GS was registered as being absent or present. Tenosynovitis was defined as hypoechoic or anechoic thickened tissue with or without fluid within the tendon sheath, which is seen in 2 perpendicular planes according to OMERACT definitions [12, 13]. Paratenonitis was identified as an echo-poor halo around the tendon in a cross-sectional scan, which often shows increased vascularity by Doppler imaging [33].

As healthy individuals often present grade 1 synovitis in GS in ultrasound studies [34, 35], the following scores were considered pathologic in our study: grades 2 and 3 for GS synovitis, grades 1-3 for PD activity, 1 (present) for GS tenosynovitis/paratenonitis, and grades 1-3 for PD tenosynovitis/paratenonitis.

In the meantime, an OMERACT score with grades 0-3 for tenosynovitis [36] was published. At the time of data collection for present study, this score was not yet published, and there were no defined scoring methods on tenosynovitis. This is the reason why tenosynovitis/paratenonitis was only scored as present or absent in this study in greyscale.

Furthermore, the US7 score later included dorsal scans of the MCP and PIP joints for GS synovitis, but this modification of the US7 score had not been performed at the time of data collection of the present study and could therefore not be used.

### Clinical and laboratory assessment

The clinical assessment included the 28 tender and swollen joint count, patient’s global assessment (PGA) of disease activity on a visual analog scale (VAS 0-100 mm), C-reactive protein (CRP; mg/L) and erythrocyte sedimentation rate (ESR; mm/h) at each visit, while IgM rheumatoid factor (IgM-RF; U/ml) and antibodies against citrullinated peptides (ACPA; U/ml) levels were only assessed at baseline. The 28-joint disease activity score (DAS28) was calculated.

### Early and established RA

To investigate the impact of disease duration on the performance of the US7 score, we divided the analysis population into two subgroups: group 1 with early RA (eRA) and a disease duration of ≤ 2 years and group 2 with established RA (estRA) and a disease duration of > 2 years.

### Statistical analysis

In a descriptive analysis, we firstly determined the frequency of pathologic joint/tendon regions (including palmar and dorsal side) at baseline and after 3 and 6 months.

Differences between the palmar and the dorsal sides of the included PIP2,3 and MCP2,3 for PD synovitis and differences between the flexor and extensor tendons for tenosynovitis/paratenonitis were analyzed using the chi-square test.

To analyze the joint/tendon regions (including palmar and dorsal sides) most sensitive to change under therapy within 6 months, the gradings of the US-joint inflammation were compared between baseline, 3 months and 6 months by Friedman test with Dunn test as post-hoc test. When a significant change over time was confirmed, we applied Bonferroni correction for multiple testing. For dichotomous measurements, comparisons were carried out by Cochrane-Q test with McNemar test as post-hoc test.

Values of < 0.05 were considered to indicate significance.

Based on the results of the descriptive analyses, we chose the joint/tendon regions that were most frequently affected by synovitis and tenosynovitis/paratenonitis and individually changed significantly during therapy for different combinations of a reduced (US7) score, separately for GS and PD. For these combinations, we calculated the standardized response means (SRM) to test their responsiveness. The SRMs with 95% confidence interval were calculated by bootstrapping with 5000 replications after 3 and 6 months. SRM was defined as mean change/standard deviation of the change. These analyses were performed separately for GS and PD. Furthermore, we calculated the SRM for DAS28 after 3 and 6 months using the same method.

The threshold values for effect sizes suggested by Jacob Cohen were used to interpret the magnitude of the SRM and values above 0.20, 0.50, and 0.80 represent small, moderate, and large responsiveness, respectively [37].

Additionally, we used linear regression to quantify the amount of information that the reduced scores retained from the original US7 score.

To assess the proportion of total information retained by the several predefined scores at baseline, univariable linear regression analyses were performed with the total joint/tendon score as the dependent variable and the reduced scores as independent variable. The corrected *R*^2^ reflected the proportion of information in the total GS/PD score retained by the selected combinations, meaning that the higher the score, i.e. the closer to 1, the better. The regression analyses were performed separately for GS and PD.

As a subanalysis, we repeated the analyses for early RA and established RA separately. Differences between the groups were analyzed using the chi-square test and Fisher’s exact test. Statistical analysis was performed using SPSS statistical software version 25.0.

## Results

### Analysis population and baseline characteristics

Four hundred thirty-five patients (80.2% female) with RA were included. At inclusion, mean (SD) age was 56.5 (SD 13.1) years, disease duration 8.2 (9.1) years, BMI 26.5 (5.1), and DAS28 4.70 (1.39). Regarding the laboratory assessment mean (SD), ESR was 28.9 (20.7), CRP 16.2 (22.1), 69% RF-IgM positivity, and 66% ACPA positivity. Of the included patients, 138 (32%) had early RA (eRA) (see Table 1).

**Table 1:** Patients’ characteristics at baseline

Parameter	All (***n*** = 435)	Early RA (***n*** = 138)	Established RA (***n*** = 297)	***p***-value
**Age [years]#**	56.5 ± 13.1* (19-83)	55.5 ± 12.9* (19-83)	57.0 ± 13.3* (19-83)	0.209^c^
**Sex (female) [%]**	80.2 %* (349)	72.5%* (100)	83.8%* (249)	0.009^a^
**BMI [kg/m**^**2**^**]#**	26.5 ± 5.1** (16-52)	26.5 ± 4.9** (17-45)	26.5 ± 5.2* (16-52)	0.825^c^
**Disease duration [years]#**	8.2 ± 9.1*(0-58.3)	0.9 ± 0.6* (0-2)	11.6 ± 9.2* (2.1-58.3)	
**DAS28#**	4.70 ± 1.39** (1-8)	4.75 ± 1.39** (2-8)	4.67 ± 1.40** (1-8)	0.473^c^
**ESR [mm/h]#**	28.9 ± 20.7* (1-115)	30.2 ± 22.0* (2-88)	28.3 ± 20.1** (1-115)	0.575^c^
**CRP [mg/l]#**	16.2 ± 22.1*** (0-162)	20.4 ± 29.4*** (0-162)	14.2 ± 17.4*** (0-120)	0.249^c^
**RF (positive) [%]**	69.2%* (301)	58.7%* (81)	74.1%* (220)	0.004^a^
**ACPA (positive) [%]**	66.0%** (287)	64.5%* (89)	66.7% **(198)	0.395^a^

Baseline characteristics for eRA (early RA) and estRA (established RA)

^#^Mean ± SD (range); * < 1% missing; ** < 5% missing, ***< 15% missing; ^c^ Mann-Whitney *U* test; ^a^chi-square test; *ESR* erythrocyte sedimentation rate, *CRP* C reactive protein, *DAS28* Disease Activity Score in 28 joints, *RF* IgM rheumatoid factor, *ACPA* anti-cyclic citrullinated peptide antibodies

### Frequency of pathologic joint/tendon regions

Palmar MCP2 and the dorsal wrist were most frequently affected by synovitis in GS (35% and 45%, respectively) and PD mode (28% and 43%) at baseline. The least affected by synovitis were PIP2 and PIP3 in GS (10% and 15%) and PD mode (PIP2 dorsal 6%, palmar 11%; PIP3 dorsal 6%, palmar 9%).

Tenosynovitis was most frequently found in the EDC and ECU tendon in GS (30%/25%) and PD mode (11%/11%) at baseline (Table 2).

**Table 2:** Pathologic joint/tendon regions at baseline with comparison of the dorsal vs. palmar sides

	**Synovitis in GS (score > = 2)**	
**Joint regions**	**All (*n*** **= 435)**	***p*-value (comparison of joint sides)**
Wrist dorsal	**44.8% (195)**	< 0.001ª (dorsal > palmar, dorsal > ulnar)
Wrist palmar	30.1% (131)	
Wrist ulnar	34.5% (150)	
MCP2 palmar	**34.5% (150)**	-
MCP3 palmar	23.4% (102)	-
PIP2 palmar	10.1% (44)	-
PIP3 palmar	14.9% (65)	-
MTP2 dorsal	25.1% (109)	-
MTP5 dorsal	17.0% (74)	-
	**Synovitis in PD (score > = 1)**	
Wrist dorsal	**43.0% (187)**	< 0.001ª (dorsal > palmar, dorsal > ulnar)
Wrist palmar	26.9% (117)	
Wrist ulnar	30.1% (131)	
MCP2 dorsal	**18.4% (80)**	< 0.001^a^ (palmar > dorsal)
MCP2 palmar	**27.8% (121)**	
MCP3 dorsal	13.3% (58)	< 0.001^a^ (palmar > dorsal)
MCP3 palmar	18.6% (81)	
PIP2 dorsal	6.0% (26)	< 0.001^a^ (palmar > dorsal)
PIP2 palmar	10.8% (47)	
PIP3 dorsal	6.2% (27)	< 0.001^a^ (palmar > dorsal)
PIP3 palmar	9.4% (41)	
MTP2 dorsal	13.8% (60)	-
MTP5 dorsal	11.0% (48)	-
	**Tenosynovitis/paratenonitis in GS (score = 1)**	
	**All (*n*= 435)**	***p*-value (comparison of tendons)**
EDC	**30.1% (131)**	< 0.001ª (EDC > FDS/P, EDC > ECU)
FDS/P	20.9% (91)	
ECU	**24.8% (108)**	
ET2	12.6% (55)	< 0.001^a^ (FT > ET)
FT2	18.4% (80)	
ET3	10.8% (47)	< 0.001^a^ (FT > ET)
FT3	14.7% (64)	
	**Tenosynovitis/paratenonitis in PD (score > = 1)**	
EDC	**11.0% (48)**	< 0.001ª (EDC > FDS/P, EDC > ECU)
FDS/P	7.8% (34)	
ECU	**10.6% (46)**	
ET2	3.9% (17)	< 0.001^a^ (FT > ET)
FT2	4.8% (21)	
ET3	3.2% (14)	< 0.001^a^ (FT > ET)
FT3	4.4% (19)	

Pathologic joint/tendon regions at baseline with comparison of the dorsal vs. palmar sides

^a^Chi-square test, *MCP* metacarpophalangeal, *PIP* proximal interphalangeal, *MTP* metatarsophalangeal, *FT/ET2* flexor/extensor tendon on MCP2 level, *FT/ET3* flexor/extensor tendon on MCP 3 level, *FDS/P* flexor digitorum superficialis/profundus tendon, *EDC* extensor digitorum communis tendon (extensor compartment lV), *ECU* extensor carpi ulnaris tendon (extensor compartment Vl), in bold: joint/tendon regions used in the reduced (US7) score

### Differences between the dorsal and palmar joint/tendon sides

At baseline, the dorsal side of the wrist was more frequently affected by synovitis than the palmar side (GS dorsal: 45%, palmar: 28%, *p* < 0.001; PD dorsal 43%, palmar: 27%, *p* < 0.001).

Furthermore, the palmar side of the finger joints was more frequently affected by synovitis than the dorsal side (e.g., for MCP2 dorsal 18%, palmar 28%, *p* < 0.001 in PD).

Moreover, the extensor tendons of the wrist were more frequently affected than the flexor tendons of the wrist (*p* < 0.001), and the flexor tendons of the fingers were more frequently affected than the extensor tendons of the fingers (*p* < 0.001) (Table 2). The same could be observed after 3 and 6 months (data not shown).

### Responsiveness to therapy

Synovitis in the wrist regions (palmar, dorsal, and ulnar side in GS mode as well as dorsal and ulnar side in PD mode) and in the palmar side of MCP2, MCP3, PIP3, and dorsal MTP5 in GS showed a significant improvement already after 3 months of therapy (T0-T1). All examined joint regions showed treatment response after 6 months (T0-T2).

After 3 months (T0-T1), all tendons showed improvement in GS, but not in PD mode. All examined tendons except FT2 in PD (*p* = 0.390) showed therapy response after 6 months (Table 3).

**Table 3:** Change after 3 and 6 months under therapy, all patients

Joint	Region	Mode	***n***	***p***-value		
				Baseline to 3 months (T0-T1)	3 to 6 months (T1-T2)	Baseline to 6 months (T0-T2)
**Synovitis**						
**Wrist**	Dorsal	GS	401	< 0.001^b^	1.000^b^	< 0.001^a^
	Palmar	GS	398	0.022^b^	0.770^b^	< 0.001^a^
	Ulnar	GS	393	< 0.001^b^	1.000^b^	< 0.001^a^
	Dorsal	PD	354	0.002^b^	1.000^b^	< 0.001^a^
	Palmar	PD	348	0.146^b^	1.000^b^	< 0.001^a^
	Ulnar	PD	343	0.010^b^	1.000^b^	< 0.001^a^
**MCP2**	Palmar	GS	401	0.008^b^	0.574^b^	< 0.001^a^
	Dorsal	PD	286	0.121^b^	1.000^b^	< 0.001^a^
	Palmar	PD	349	0.093^b^	0.635^b^	< 0.001^a^
**MCP3**	Palmar	GS	397	0.004^b^	1.000^b^	< 0.001^a^
	Dorsal	PD	283	0.424^b^	1.000^b^	< 0.001^a^
	Palmar	PD	343	0.326^b^	0.991^b^	< 0.001^a^
**PIP2**	Palmar	GS	399	0.058^b^	1.000^b^	< 0.001^a^
	Dorsal	PD	250	0.943^b^	1.000^b^	< 0.001^a^
	Palmar	PD	325	0.364^b^	1.000^b^	< 0.001^a^
**PIP3**	Palmar	GS	397	0.030^b^	1.000^b^	< 0.001^a^
	Dorsal	PD	253	0.799^b^	1.000^b^	< 0.001^a^
	Palmar	PD	324	1.000^b^	1.000^b^	0.002^a^
**MTP2**	Dorsal	GS	392	0.131^b^	1.000^b^	0.002^a^
	Dorsal	PD	303	0.313^b^	1.000^b^	< 0.001^a^
**MTP5**	Dorsal	GS	390	0.033^b^	1.000^b^	< 0.001^a^
	Dorsal	PD	296	0.565^b^	1.000^b^	< 0.001^a^
**Tenosynovitis/paratenonitis**						
**Wrist**	EDC	GS	392	< 0.001^d^	1.000^d^	< 0.001^c^
	EDC	PD	299	0.891^d^	1.000^d^	0.003^c^
	FDS/P	GS	393	< 0.001^d^	1.000^d^	< 0.001^c^
	FDS/P	PD	295	1.000^d^	1.000^d^	0.041^c^
	ECU	GS	391	0.002^d^	0.741^d^	< 0.001^c^
	ECU	PD	295	0.853^d^	1.000^d^	0.001^c^
**MCP level**	ET2	GS	393	< 0.001^d^	0.805^d^	< 0.001^c^
	ET2	PD	259	1.000^d^	1.000^d^	< 0.001^c^
	ET3	GS	391	< 0.001^d^	1.000^d^	< 0.001^c^
	ET3	PD	257	1.000^d^	1.000^d^	0.012^c^
	FT2	GS	392	< 0.001^d^	0.333^d^	< 0.001^c^
	FT2	PD	248	-	-	0.390^c^
	FT3	GS	384	< 0.001^d^	0.715^d^	< 0.001^c^
	FT3	PD	242	1.000^d^	1.000^d^	0.015^c^

*P*-values depicting change of severity (grades) over time (the underlying data incl. effect sizes can be found in supplementary tables 3.1 and 3.2)

^a^Friedman test, ^b^Dunn test as post-hoc test, ^c^Cochrane-*Q* test (GS) and Friedman test (PD) resp.; ^d^McNemar test (GS) and Dunn test (PD) as post-hoc tests resp.; *GS* greyscale, *PD* power Doppler

*MCP* metacarpophalangeal, *PIP* proximal interphalangeal, *MTP* metatarsophalangeal, *FT/ET2* flexor/extensor tendon on MCP2 level, *FT/ET3* flexor/extensor tendon on MCP3 level, *FDS/P* flexor digitorum superficialis/profundus tendon, *EDC* extensor digitorum communis tendon (extensor compartment lV), *ECU* extensor carpi ulnaris tendon (extensor compartment Vl)

Based on the results of the analyses above and according to expert agreement, we defined possible joint region/tendon combinations as possible reduced scores, choosing the ones most frequently affected and responsive to therapy (GS 1-5 and PD 1-9) (Table 4), e.g. the dorsal wrist and the palmar MCP2 were a part of all of the possible combinations as they showed to be most frequently affected out of all joint regions.

**Table 4:** Joint/tendon combinations as possible scores

Mode	Combination	Included joint/tendon regions
GS	GS1	Dorsal wrist, palmar MCP2
	**GS2**	**Dorsal wrist, palmar MCP2, EDC, ECU**
	GS3	Dorsal wrist, palmar MCP2, palmar MCP3
	GS4	Dorsal wrist, palmar MCP2, palmar MCP3, EDC, ECU
	GS5	Dorsal wrist, palmar MCP2, palmar MCP3, EDC, ECU, FT2, FT3
PD	PD1	Dorsal wrist, palmar MCP2
	PD2	Dorsal wrist, palmar MCP2, EDC, ECU
	PD3	Dorsal wrist, palmar MCP2, dorsal MCP2
	PD4	Dorsal wrist, palmar MCP2, palmar MCP3
	PD5	Dorsal wrist, palmar MCP2, palmar MCP3, EDC, ECU
	PD6	Dorsal wrist, palmar MCP2, palmar MCP3, dorsal MCP2, dorsal MCP3
	PD7	Dorsal wrist, palmar MCP2, palmar MCP3, dorsal MCP2, dorsal MCP3, EDC, ECU tendons
	PD8	Dorsal wrist, palmar MCP2, palmar MCP3, dorsal MCP2, dorsal MCP3, EDC, ECU, FT2, FT3
	**PD9**	**Dorsal wrist, palmar MCP2, dorsal MCP2, EDC, ECU**

*GS* greyscale, *PD* power Doppler, *MCP* metacarpophalangeal, *PIP* proximal interphalangeal, *MTP* metatarsophalangeal, *FT/ET2* flexor/extensor tendon on MCP2 level, *FT/ET3* flexor/extensor tendon on MCP3 level, *FDS/P* flexor digitorum superficialis/profundus tendon, *EDC* extensor digitorum communis tendon (extensor compartment lV), *ECU* extensor carpi ulnaris tendon (extensor compartment Vl). Combinations in bold were included in the reduced score

**Fig. 1:**
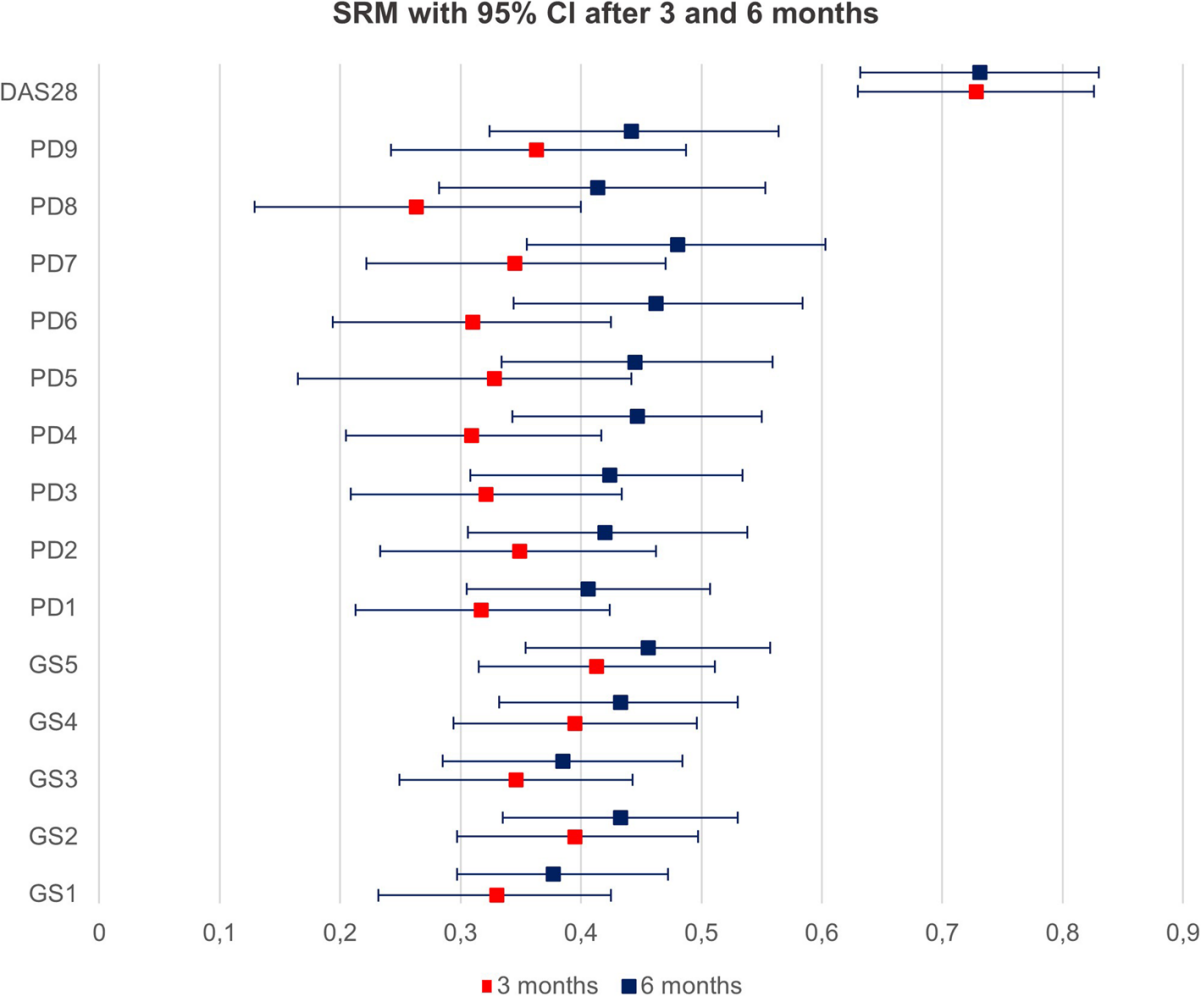
SRM with 95% CI for possible score combinations and DAS28 after 3 and 6 months

The combination GS2 (dorsal wrist, palmar MCP2, EDC, ECU tendons) showed a moderate sensitivity to change after 3 and 6 months (0.40 and 0.44) while retaining 69% (GS) of the full US7 score’s information (see Fig. 1 and Table 5).

**Table 5:** Proportion of the US7 score’s information retained by different joint/tendon combinations

Mode	Joint/tendon combination	All		Early RA		Established RA	
		***n***	Corrected ***R***^**2**^	***n***	Corrected ***R***^**2**^	***n***	Corrected ***R***^**2**^
**GS**	GS1	420	0.596	131	0.576	289	0.604
	**GS2**	**410**	**0.686**	**128**	**0.701**	**282**	**0.686**
	GS3	420	0.627	131	0.628	289	0.628
	GS4	410	0.712	128	0.739	282	0.704
	GS5	399	0.748	127	0.775	272	0.736
**PD**	PD1	375	0.588	116	0.682	259	0.570
	PD2	331	0.732	99	0.784	232	0.712
	PD3	316	0.652	99	0.824	217	0.646
	PD4	373	0.653	116	0.721	257	0.618
	PD5	330	0.750	99	0.818	231	0.722
	PD6	313	0.711	97	0.872	216	0.665
	PD7	293	0.837	89	0.932	204	0.771
	PD8	256	0.868	70	0.950	186	0.813
	**PD9**	**295**	**0.785**	**91**	**0.856**	**204**	**0.735**
**GS/PD**	**GS2 + PD9**	**290**	**0.756**	**89**	**0.855**	**201**	**0.727**

The corrected *r*^2^ delivered the proportion of information in the total US7 GS score/PD score retained by the selected combinations. Combinations: see Table 4; combinations in bold were included in the reduced score; *GS* greyscale Scale, *PD* power Doppler

Also, the combination PD9 (dorsal wrist, palmar MCP2, dorsal MCP2, EDC, ECU) depicted a low to moderate sensitivity to change after 3 and 6 months (0.36 and 0.44) with a reduced number of joint/tendon regions while retaining 79% (PD) of the full US7 score’s information (Table 5).

For comparison purposes, the SRM for DAS28 after 3 and 6 months was good: 0.728 after 3 months and 0.731 after 6 months.

**Fig. 2:**
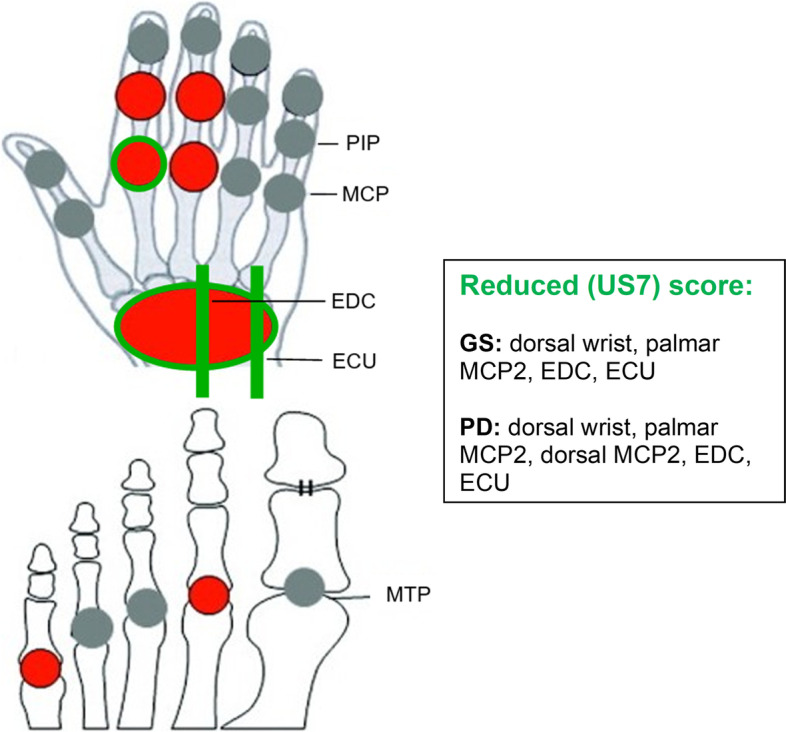
Reduced (US7) score. In red: joint regions included in the original US7 score; in green: reduced (US7) score; illustration adapted from Backhaus et al. [19]; *MCP* metacarpophalangeal, *PIP* proximal interphalangeal, *MTP* metatarsophalangeal, *EDC* extensor digitorum communis tendon (extensor compartment lV), *ECU* extensor carpi ulnaris tendon (extensor compartment Vl)

The reduced (US7) score that performed the best (i.e. being sensitive to change and retaining most information of the original score while requiring the lowest number of items possible) includes the dorsal wrist, palmar MCP2, EDC and ECU tendons in GS mode and the dorsal wrist, palmar MCP2, dorsal MCP2, EDC and ECU tendons in PD mode (Fig. 2). This combination showed to be sensitive to change (SRM 0.433), requiring the lowest number of items (*n* = 4) to be assessed while retaining most of the US7 score’s information (i.e. 75%).

### Early versus established RA

Few significant differences between early and established RA could be detected in single joint regions regarding the frequency of pathologic joint regions. For instance, at baseline, PD of dorsal wrist and palmar PIP2 were more frequently pathologic in the group of established RA (Supplementary Table 1).

After 3 months, the palmar wrist and palmar MCP2 were more frequently pathologic in GS in the group of established RA (*p* = 0.016 and *p* = 0.006, respectively) (data not shown). In both groups, most joint regions were responsive to therapy and only few regions performed worse in the group of established RA; these were PD synovitis of the palmar wrist from baseline to 3 months (T0-T1, *p* = 0.050), GS tenosynovitis of EDC tendon of the wrist from baseline to 3 months (T0-T1, *p* = 0.038), and GS tenosynovitis of the flexor tendon of the wrist from baseline to 3 months (T0-T1, *p* = 0.008) (data not shown).

**Fig. 3:**
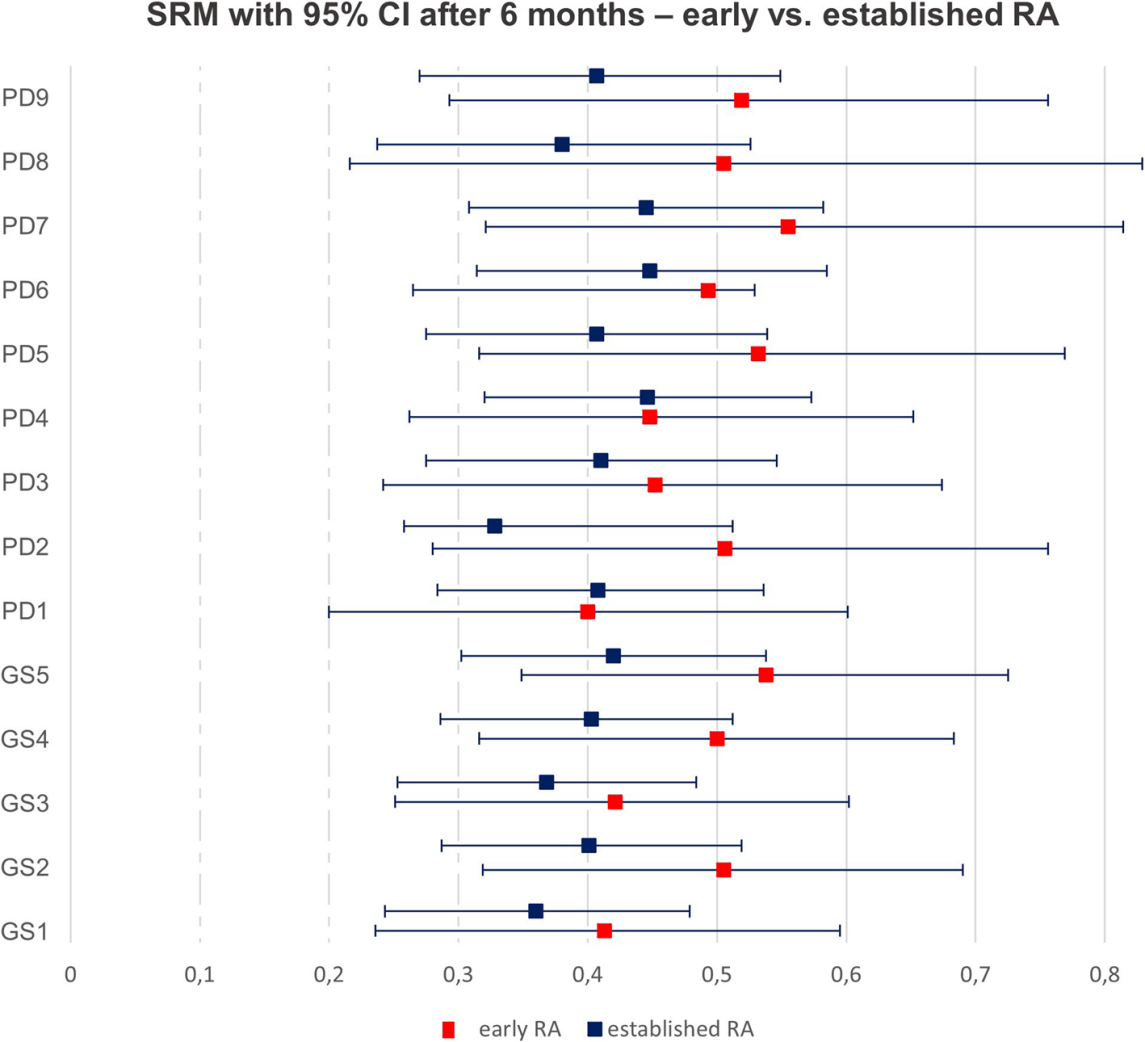
SRM with 95% CI after 6 months - early vs. established RA

Regarding the calculated SRMs, the combinations that performed the best, i.e. GS2 and PD9, depicted a slightly higher sensitivity to change in the group of early versus established RA after 3 months (SRM GS2: 0.55 vs. 0.32 and PD9: 0.44 vs. 0.33) and 6 months (SRM GS2: 0.505 vs. 0.401 and PD9: 0.519 vs. 0.407) (Supplementary Table 4/Fig. 3). The retained information obtained about the same level for both groups (GS2: 70% for eRA vs. 69% for estRA and PD9 86% for eRA vs. 73% for estRA) (Table 5).

## Discussion

In the present study, we reassessed the US7 score and found the dorsal wrist, the second MCP as well as EDC and ECU tendons to be most frequently pathologic and responsive to therapy. Following GS and PD combination of joints/tendons performed well as reduced (US7) score, being responsive to therapy while retaining most of the previous information of the original US7 score: GS and PD of the dorsal wrist, palmar MCP2, extensor digitorum communis and extensor carpi ulnaris tendons plus PD of dorsal MCP2. The reduced number of items (*n* = 4) also reduces the time needed for examination, thus making it more feasible than the original US7 score. The exact time needed to execute the reduced score is estimated to be around 3 to 5 min from clinical experience. We excluded MTP2 and MTP5, as these joint regions were not as frequently affected and less responsive to therapy (only after 6 months, not already after 3 months). Furthermore, previous studies have shown that GS pathology in MTP joints is common in healthy individuals [34, 35] and may not be specific for rheumatoid arthritis.

Moreover, the patients were recruited already between 2006 and 2010 [19, 27] when the definition of synovitis included effusion next to synovial hypertrophy, which is not anymore included in the recently published EULAR/OMERACT recommendations [17]. Aga et al. previously developed the ‘USRA9’ score based on a data driven approach. The score performed better than several other joint scores including the US7 score [21]. The USRA9 score also includes the wrist, the ECU tendon and MCP2, but it only examines the dorsal joint regions. Our study showed that in PD mode the finger joints were more frequently affected by synovitis at the palmar joint side. This aspect is therefore missing in the USRA9 score.

In our study, the wrist and MCP2 were examined from both sides in PD mode and the wrist from both sides in GS mode. MCP2 as well as MCP3, PIP2 and PIP3 have only been examined from palmar in GS mode, not from dorsal, so that in the present study, we could not compare the dorsal and palmar side of the finger joints in GS mode, only in PD mode. Later, the US7 score has been further developed to include the examination of both palmar and dorsal joint sides both for PD and GS.

Vlad et al. [38] as well as Scheel et al. [32] found the palmar side of MCP and PIP joints to be more frequently affected by synovitis than the dorsal side. We also found the palmar sides of the MCP and PIP joints in PD to be more frequently affected by synovitis. Thus, palmar examination of the finger joints should be included in an optimal ultrasound score for RA.

Furthermore, only the most affected hand/foot has been examined in our study in contrast to the USRA9 score including bilateral examination. The US7 score was developed according to RAMRIS [39] which showed the same results when only the clinically dominant hand (instead of both hands) was examined to detect disease activity in RA. We concluded that, without losing crucial information, an unilateral examination saves time in daily practice, improving feasibility.

Further studies on whether to scan the dorsal or palmar side are needed, as our study lacks the examination of both sides in GS mode.

The definition of paratenonitis according to Grassi et al. [33] of the extensor tendons of MCP2,3 was used in the first publication on the US7 score as the extensor tendons of MCP2,3 were thought to have no tendon sheath. In contrast to that, just recently, a publication by Dakkak et al. [40] showed that the extensor tendons do have a tendon sheath. In our study, we compared the frequency of tenosynovitis of the extensor tendon vs. flexor tendon at the level of MCP2,3 and found that the flexor tendons were more frequently affected, independently from RA disease duration.

Several reduced ultrasound scores using different methods to identify essential joints to be included have been published, like the twelve-joint score by Naredo et al. [30], the 6 -joint score by Perricone et al. [20], the US10 Score by Luz et al. [24], and the eight-joint score by Yoshimi et al. [41]. All of them included the wrist and MCP2 as they counted to the joint regions that were most frequently affected and also responsive to therapy in data driven approaches, which is also supported by the data of our study. Therefore, these two joints and the EDC and ECU tendons are-according to the results of this analysis-essential to be included in an optimal scoring system.

Based on this, we have investigated the performance of reduced (US7) scores, among which a score including the dorsal wrist, MCP2, EDC, and ECU showed SRMs around 0.4. Other scores, such as the USRA9, had higher SRMs but also included more joint regions, resulting in a longer scanning duration. A lower SRM could be accounted for by a smaller number of parameters included in the SRM analysis. The SRM calculated for DAS28 at 3 and 6 months was around 0.7 in our study, showing a good sensitivity to change of the clinical response, as could be expected because of combination of several variables. However, the DAS28 has a subjective aspect including the patient’s global score on disease activity. The added value of ultrasound examination is its ability to objectify joint pain and visualize inflammation as well as the severity of inflammation. It is therefore an important tool for therapeutic decisions and the monitoring of therapy.

We also investigated the impact of disease duration (early vs. established RA) on the performance of the US7 score to explore if the score is appropriate to be used at all stages of disease. No major differences were found when analyzing the individual joint or tendon regions. Concerning the reduced (US7) score, its performance was slightly better in patients with early RA regarding the responsiveness and at about the same level concerning the percentage of information retained from the original US7 score. In this analysis we found no major impact of the disease duration on the performance of the score.

## Conclusions

To summarize, the MCP2, the dorsal wrist and extensor compartments IV and VI were the joint/tendon regions most frequently affected and responsive to therapy. A reduced score including those joint and tendon regions is sensitive to change and should be very feasible (3-5 min. examination time), especially in daily practice of a rheumatologist. Therefore, a global composite ultrasound score for therapy monitoring of patients with RA should at least include the dorsal wrist and MCP2 as well as extensor compartments IV and VI.

## Supplementary Information

**Additional file 1: Supplementary Table 1**: Pathologic joint/tendon regions at baseline – comparison of the groups. **Supplementary Table 2**: Significant differences between eRA and estRA in frequency of affected joint/tendon regions during the study. **Supplementary Table 3.1**: Synovitis at baseline, 3 and 6 months; change of synovitis, all (*n* = 435). **Supplementary Table 3.2**: Tenosynovitis/paratenonitis at baseline, 3 and 6 months, change of tenosynovitis/paratenonitis, all (*n* = 435). **Supplementary Table 4**: SRM with 95% Cl after 3 months, 3 to 6 and 6 months.

## Data Availability

The datasets used and/or analyzed during the current study are available from the corresponding author on reasonable request.

## Abbreviations

ACR: American College of Rheumatology
ACPA: Anti-cyclic citrullinated peptides
BDMARD: Biological disease-modifying anti-rheumatic drug
CRF: Case report form
CRP: C-reactive protein
CsDMARD: Conventional synthetic disease-modifying anti-rheumatic drug
DAS28: Disease activity score of 28 joints
ECU: Extensor carpi ulnaris tendon (extensor compartment VI)
EDC: Extensor digitorum communis tendon (extensor compartment IV)
ESR: Erythrocyte sedimentation rate
ET: Extensor tendon
EULAR: European alliance of associations for rheumatology
FDS/P: Flexor digitorum superficialis/profundus tendon
FT: Flexor tendon
GS: Greyscale (ultrasound)
IgM-RF: Immunoglobulin M-rheumatoid factor
MCP: Metacarpophalangeal (joint)
MRI: Magnetic resonance imaging
MSUS: Musculoskeletal ultrasound
MTP: Metatarsophalangeal (joint)
NSAIDs: Non-steroidal anti-inflammatory drugs
OMERACT: Outcome measures in rheumatology clinical trial
PIP: Proximal interphalangeal (joint)
PD: Power Doppler (ultrasound)
RA: Rheumatoid arthritis
SRM: Standardized response means
TNF: Tumor necrosis factor
TsDMARD: Targeted synthetic disease-modifying anti-rheumatic drug
US: Ultrasound
