# Supplementary material for: The MCP2 and the wrist plus two extensor compartments are the most affected and responsive joints/tendons out of the US7 score in patients with rheumatoid arthritis-an observational study
Source: Arthritis Res Ther. 2022 Aug 5;24:183. doi: 10.1186/s13075-022-02874-y (PMC9354335; doi:10.1186/s13075-022-02874-y)
Supplement: Supplementary file 1 — Additional file 1: Supplementary Table 1: Pathologic joint/tendon regions at baseline – comparison of the groups. Supplementary Table 2: Significant differences between eRA and estRA in frequency of affected joint/tendon regions during the study. Supplementary Table 3.1: Synovitis at baseline, 3 and 6 months; change of synovitis, all (n = 435). Supplementary Table 3.2: Tenosynovitis/paratenonitis at baseline, 3 and 6 months, change of tenosynovitis/paratenonitis, all (n = 435). Supplementary Table 4: SRM with 95% Cl after 3 months, 3 to 6 and 6 months. [file 13075_2022_2874_MOESM1_ESM.docx]

**Supplement**

**The MCP2 and the wrist plus two tendons** **are the most affected and responsive joints/tendons out of the US7 score in patients with rheumatoid arthritis –** **an observational study**

**Supplementary Table 1: Pathologic joint/tendon regions at baseline – comparison of the groups**

| **Synovitis in GS (score >=2)** | | | | |
| --- | --- | --- | --- | --- |
| **Joint regions** | **All (n = 435)** | **Early RA (n = 138)** | **Established RA (n = 297)** | **P-value (comparison of groups)** |
| Wrist dorsal | **44.8% (195)** | 41.3% (57) | 46.5% (138) | p = 0.314a |
| Wrist palmar | 30.1% (131) | 31.2% (43) | 29.6% (88) | p = 0.746a |
| Wrist ulnar | 34.5% (150) | 31.2% (43) | 36.0% (107) | p = 0.320a |
| MCP2 palmar | **34.5% (150)** | 32.6% (45) | 35.4% (105) | p = 0.575a |
| MCP3 palmar | 23.4% (102) | 21.7% (30) | 24.2% (72) | p = 0.566a |
| PIP2 palmar | 10.1% (44) | 8.7% (12) | 10.8% (32) | p = 0.503a |
| PIP3 palmar | 14.9% (65) | 15.2% (21) | 14.8% (44) | p = 0.913a |
| MTP2 dorsal | 25.1% (109) | 27.5% (38) | 23.9% (71) | p = 0.416a |
| MTP5 dorsal | 17.0% (74) | 16.7% (23) | 17.2% (51) | p = 0.896a |
| **Synovitis in PD (score >=1)** | | | | |
| Wrist dorsal | **43.0% (187)** | 35.5% (49) | 46.5% (138) | p = 0.032a |
| Wrist palmar | 26.9% (117) | 27.5% (38) | 26.6% (79) | p = 0.837a |
| Wrist ulnar | 30.1% (131) | 32.6% (45) | 29.0% (86) | p = 0.440a |
| MCP2 dorsal | **18.4% (80)** | 15.2% (21) | 19.9% (59) | p = 0.224a |
| MCP2 palmar | **27.8% (121)** | 26.8% (37) | 28.3% (84) | p = 0.750a |
| MCP3 dorsal | 13.3% (58) | 10.9% (15) | 14.5% (43) | p = 0.303a |
| MCP3 palmar | 18.6% (81) | 17.4% (24) | 19.2% (57) | p = 0.653a |
| PIP2 dorsal | 6.0% (26) | 3.6% (5) | 7.1% (21) | p = 0.195b |
| PIP2 palmar | 10.8% (47) | 6.5% (9) | 12.8% (38) | p = 0.050a |
| PIP3 dorsal | 6.2% (27) | 5.1% (7) | 6.7% (20) | p = 0.504a |
| PIP3 palmar | 9.4% (41) | 8.0% (11) | 10.1% (30) | p = 0.479a |
| MTP2 dorsal | 13.8% (60) | 10.9% (15) | 15.2% (45) | p = 0.228a |
| MTP5 dorsal | 11.0% (48) | 8.7% (12) | 12.1% (36) | p = 0.289a |
| **Tenosynovitis/Paratenonitis in GS (score = 1)** | | | | |
|  | **All (n = 435)** | **eRA (n = 138)** | **estRA (n = 297)** | **P-value (comparison of groups)** |
| EDC | **30.1% (131)** | 35.5% (49) | 27.6% (82) | p = 0.095a |
| FDS/P | 20.9% (91) | 23.2% (32) | 19.9% (59) | p = 0.428a |
| ECU | **24.8% (108)** | 25.4% (35) | 24.6% (73) | p = 0.860a |
| ET2 | 12.6% (55) | 12.3% (17) | 12.8% (38) | p = 0.889a |
| FT2 | 18.4% (80) | 21.0% (29) | 17.2% (51) | p = 0.336a |
| ET3 | 10.8% (47) | 9.4% (13) | 11.4% (34) | p = 0.526a |
| FT3 | 14.7% (64) | 18.1% (25) | 13.1% (39) | p = 0.172a |
| **Tenosynovitis/Paratenonitis in PD (score >= 1)** | | | | |
| EDC | **11.0% (48)** | 11.6% (16) | 10.8% (32) | p = 0.800a |
| FDS/P | 7.8% (34) | 8.7% (12) | 7.4% (22) | p = 0.641a |
| ECU | **10.6% (46)** | 13.0% (18) | 9.4% (28) | p = 0.254a |
| ET2 | 3.9% (17) | 2.9% (4) | 4.4% (13) | p = 0.599b |
| FT2 | 4.8% (21) | 2.9% (4) | 5.7% (17) | p = 0.238b |
| ET3 | 3.2% (14) | 2.2% (3) | 3.7% (11) | p = 0.563b |
| FT3 | 4.4% (19) | 2.9% (4) | 5.1% (15) | p = 0.450b |

Pathologic joint regions at baseline with comparison of the groups early RA vs. established RA and comparison of the joint planes (dorsal vs. palmar)

*a = Chi-square test, b = Fisher’s exact test, GS = greyscale, PD = power Doppler, MCP = metacarpophalangeal, PIP = proximal interphalangeal, MTP = metatarsophalangeal, FT/ET2 = flexor/extensor tendon on MCP2 level, FT/ET3 = flexor/extensor tendon on MCP3 level, FDS/P = flexor digitorum superficialis/profundus tendon, EDC = extensor digitorum communis tendon (extensor compartment lV), ECU = extensor carpi ulnaris tendon (extensor compartment Vl), in bold: joint/tendon regions included in reduced score*

**Supplementary Table 2: Significant differences between eRA and estRA in frequency of affected joint/tendon regions during the study**

| **Joint region,**  **Mode, visit** | **Grade** | **All (n =435)** | **Early RA (n = 138)** | **Established RA**  **(n=297)** | **P-value (comparison between the groups)** |
| --- | --- | --- | --- | --- | --- |
|  |  | **Prop. (n)** | **Prop. (n)** | **Prop. (n)** |  |
| **Dorsal wrist**  **PD**  **baseline** | 0 | 44.6% (194) | 50.0% (69) | 42.1% (125) | (p = 0.009)^a^ |
|  | 1 | 25.5% (111) | 23.9% (33) | 26.3% (78) |  |
|  | 2 | 13.3% (58) | 5.8% (8) | 16.8% (50) |  |
|  | 3 | 4.1% (18) | 5.8% (8) | 3.4% (10) |  |
|  | Missing | 12.4% (54) | 14.5% (20) | 11.4% (34) |  |
| **Dorsal MCP3**  **PD**  **baselie** | 0 | 60.5% (263) | 61.6% (85) | 59.9% (178) | (p = 0.042)b |
|  | 1 | 10.1% (44) | 9.4% (13) | 10.4% (31) |  |
|  | 2 | 2.5% (11) | 0.0% (0) | 3.7% (11) |  |
|  | 3 | 0.7% (3) | 1.4% (2) | 0.3% (1) |  |
|  | Missing | 26.2% (114) | 27.5% (38) | 25.6% (76) |  |
| **Dorsal MTP5**  **GS**  **baseline** | 0 | 58.9% (256) | 63.8% (88) | 56.6% (168) | (p = 0.020)^b^ |
|  | 1 | 20.2% (88) | 13.0% (18) | 23.6% (70) |  |
|  | 2 | 10.6% (46) | 13.0% (18) | 9.4% (28) |  |
|  | 3 | 6.4% (28) | 3.6% (5) | 7.7% (23) |  |
|  | Missing | 3.9% (17) | 6.5% (9) | 2.7% (8) |  |
| **Palmar MCP2**  **GS**  **3 months** |  | 40.2% (175) | 47.8% (66) | 36.7% (109) | (p = 0.036)^a^ |
|  | 1 | 32.6% (142) | 33.3% (46) | 32.3% (96) |  |
|  | 2 | 12.9% (56) | 8.7% (12) | 14.8% (44) |  |
|  | 3 | 11.3% (49) | 7.2% (10) | 13.1% (39) |  |
|  |  | 3.0% (13) | 2.9% (4) | 3.0% (9) |  |
| **Palmar PIP2**  **PD**  **6 months** | 0 | 78.4% (341) | 80.4% (111) | 77.4% (230) | (p = 0.037)^b^ |
|  | 1 | 5.5% (24) | 2.2% (3) | 7.1% (21) |  |
|  | 2 | 0.5% (2) | 0.0% (0) | 0.7% (2) |  |
|  | 3 | 0.2% (1) | 0.7% (1) | 0.0% (0) |  |
|  | Missing | 15.4% (67) | 16.7% (23) | 14.8% (44) |  |
| **Dorsal MTP5**  **GS**  **6 months** | 0 | 68.0% (296) | 76.1% (105) | 64.3% (191) | (p = 0.029)^b^ |
|  | 1 | 18.2% (79) | 10.9% (15) | 21.5% (64) |  |
|  | 2 | 6.7% (29) | 5.1% (7) | 7.4% (22) |  |
|  | 3 | 2.5% (11) | 2.2% (3) | 2.7% (8) |  |
|  | Missing | 4.6% (20) | 5.8% (8) | 4.0% (12) |  |

^a^ Chi-square test, ^b^ Fisher’s exact test; patients with missing values are excluded from the tests. Only differences between the groups with a p-value of < 0.05 are shown in this table

*GS = greyscale, PD = power Doppler, MCP = metacarpophalangeal, PIP = proximal interphalangeal, MTP = metatarsophalangeal, FT/ET2 = flexor/extensor tendon on MCP2 level, FT/ET3 = flexor/extensor tendon on MCP3 level, FDS/P = flexor digitorum superficialis/profundus tendon, EDC = extensor digitorum communis tendon (extensor compartment lV), ECU = extensor carpi ulnaris tendon (extensor compartment Vl)*

**Supplementary Table 3.1 Synovitis at baseline, 3 and 6 months;** **change of synovitis, all (n = 435)**

| **Joint region** | | **Grade** | **Baseline (T0)** | | **3 months (T1)** | | | | **6 months (T2)** | | **p-value** | **p-value** | **p-value** |
| --- | --- | --- | --- | --- | --- | --- | --- | --- | --- | --- | --- | --- | --- |
|  |  |  | **Prop.** | **N** | **Prop.** | | **N** | | **Prop.** | **N** | **(T0-T2)** | **(T0-T1)** | **(T1-T2)** |
| **Wrist – GS score** | | | | | | | | | | | | | |
| **Dorsal** | | 0 | 26.0% | 113 | 32.4% | | 141 | | 36.1% | 157 | < 0.001 | < 0.001 | 1.000 |
|  | | 1 | 26.4% | 115 | 34.0% | | 148 | | 33.3% | 145 |  |  |  |
|  | | 2 | 27.1% | 118 | 18.9% | | 82 | | 17.2% | 75 |  |  |  |
|  | | 3 | 17.7% | 77 | 11.7% | | 51 | | 10.6% | 46 |  |  |  |
|  | | Miss. | 2.8% | 12 | 3.0% | | 13 | | 2.8% | 12 |  |  |  |
| **Palmar** | | 0 | 37.0% | 161 | 43.4% | | 189 | | 46.9% | 204 | < 0.001 | 0.022 | 0.770 |
|  | | 1 | 29.9% | 130 | 31.0% | | 135 | | 31.5% | 137 |  |  |  |
|  | | 2 | 18.9% | 82 | 14.7% | | 64 | | 12.0% | 52 |  |  |  |
|  | | 3 | 11.3% | 49 | 7.6% | | 33 | | 6.7% | 29 |  |  |  |
|  | | Miss. | 3.0% | 13 | 3.2% | | 14 | | 3.0% | 13 |  |  |  |
| **Ulnar** | | 0 | 35.9% | 156 | 43.7% | | 190 | | 46.0% | 200 | < 0.001 | < 0.001 | 1.000 |
|  | | 1 | 26.0% | 113 | 32.0% | | 139 | | 31.5% | 137 |  |  |  |
|  | | 2 | 20.7% | 90 | 13.6% | | 59 | | 12.9% | 56 |  |  |  |
|  | | 3 | 13.8% | 60 | 7.1% | | 31 | | 6.4% | 28 |  |  |  |
|  | | Miss. | 3.7% | 16 | 3.7% | | 16 | | 3.2% | 14 |  |  |  |
| **Wrist – PD score** | | | | | | | | | | | | | |
| **Dorsal** | | 0 | 44.6% | 194 | 54.3% | | 236 | | 60.5% | 263 | < 0.001 | 0.002 | 1.000 |
|  | | 1 | 25.5% | 111 | 22.5% | | 98 | | 14.3% | 62 |  |  |  |
|  | | 2 | 13.3% | 58 | 7.8% | | 34 | | 9.2% | 40 |  |  |  |
|  | | 3 | 4.1% | 18 | 2.3% | | 10 | | 2.5% | 11 |  |  |  |
|  | | Miss. | 12.4% | 54 | 13.1% | | 57 | | 13.6% | 59 |  |  |  |
| **Palmar** | | 0 | 60.0% | 261 | 67.4% | | 293 | | 70.3% | 306 | < 0.001 | 0.146 | 1.000 |
|  | | 1 | 17.5% | 76 | 14.0% | | 61 | | 11.5% | 50 |  |  |  |
|  | | 2 | 7.6% | 33 | 4.6% | | 20 | | 3.7% | 16 |  |  |  |
|  | | 3 | 1.8% | 8 | 0.7% | | 3 | | 0.5% | 2 |  |  |  |
|  | | Miss. | 13.1% | 57 | 13.3% | | 58 | | 14.0% | 61 |  |  |  |
| **Ulnar** | | 0 | 56.8% | 247 | 66.2% | | 288 | | 67.6% | 294 | < 0.001 | 0.010 | 1.000 |
|  | | 1 | 17.5% | 76 | 14.3% | | 62 | | 14.0% | 61 |  |  |  |
|  | | 2 | 10.3% | 45 | 4.8% | | 21 | | 3.2% | 14 |  |  |  |
|  | | 3 | 2.3% | 10 | 0.7% | | 3 | | 0.7% | 3 |  |  |  |
|  | | Miss. | 13.1% | 57 | 14.0% | | 61 | | 14.5% | 63 |  |  |  |
| **MCP2 – GS score** | | | | | | | | | | | | | |
| **Palmar** | | 0 | 34.5% | 150 | 40.2% | | 175 | | 46.0% | 200 | < 0.001 | 0.008 | 0.574 |
|  | | 1 | 28.0% | 122 | 32.6% | | 142 | | 29.7% | 129 |  |  |  |
|  | | 2 | 20.0% | 87 | 12.9% | | 56 | | 12.2% | 53 |  |  |  |
|  | | 3 | 14.5% | 63 | 11.3% | | 49 | | 9.4% | 41 |  |  |  |
|  | | Miss. | 3.0% | 13 | 3.0% | | 13 | | 2.8% | 12 |  |  |  |
| **MCP2 – PD score** | | | | | | | | | | | | | |
| **Dorsal** | | 0 | 56.1% | 244 | 64.4% | | 280 | | 65.5% | 285 | < 0.001 | 0.121 | 1.000 |
|  | | 1 | 12.9% | 56 | 8.0% | | 35 | | 6.2% | 27 |  |  |  |
|  | | 2 | 4.8% | 21 | 2.8% | | 12 | | 1.8% | 8 |  |  |  |
|  | | 3 | 0.7% | 3 | 0.5% | | 2 | | 0.7% | 3 |  |  |  |
|  | | Miss. | 25.5% | 111 | 24.4% | | 106 | | 25.7% | 112 |  |  |  |
| **Palmar** | | 0 | 60.0% | 261 | 68.0% | | 296 | | 72.6% | 316 | < 0.001 | 0.093 | 0.635 |
|  | | 1 | 18.6% | 81 | 15.6% | | 68 | | 9.4% | 41 |  |  |  |
|  | | 2 | 8.3% | 36 | 3.9% | | 17 | | 3.4% | 15 |  |  |  |
|  | | 3 | 0.9% | 4 | 0.7% | | 3 | | 0.7% | 3 |  |  |  |
|  | | Miss. | 12.2% | 53 | 11.7% | | 51 | | 13.8% | 60 |  |  |  |
| **MCP3 – GS score** | | | | | | | | | | | | | |
| **Palmar** | | 0 | 42.8% | 186 | 52.0% | | 226 | | 56.1% | 244 | < 0.001 | 0.004 | 1.000 |
|  | | 1 | 30.8% | 134 | 30.3% | | 132 | | 27.8% | 121 |  |  |  |
|  | | 2 | 14.7% | 64 | 8.3% | | 36 | | 7.8% | 34 |  |  |  |
|  | | 3 | 8.7% | 38 | 5.7% | | 25 | | 5.3% | 23 |  |  |  |
|  | | Miss. | 3.0% | 13 | 3.7% | | 16 | | 3.0% | 13 |  |  |  |
| **MCP3 – PD score** | | | | | | | | | | | | | |
| **Dorsal** | | 0 | 60.5% | 263 | 67.4% | | 293 | | 70.6% | 307 | < 0.001 | 0.424 | 1.000 |
|  | | 1 | 10.1% | 44 | 5.7% | | 25 | | 2.5% | 11 |  |  |  |
|  | | 2 | 2.5% | 11 | 1.6% | | 7 | | 1.1% | 5 |  |  |  |
|  | | 3 | 0.7% | 3 | 0.5% | | 2 | | 0.2% | 1 |  |  |  |
|  | | Miss. | 26.2% | 114 | 24.8% | | 108 | | 25.5% | 111 |  |  |  |
| **Palmar** | | 0 | 68.7% | 299 | 75.9% | | 330 | | 78.9% | 343 | < 0.001 | 0.326 | 0.991 |
|  | | 1 | 13.3% | 58 | 8.5% | | 37 | | 4.4% | 19 |  |  |  |
|  | | 2 | 4.6% | 20 | 3.4% | | 15 | | 2.5% | 11 |  |  |  |
|  | | 3 | 0.7% | 3 | - | | - | | 0.2% | 1 |  |  |  |
|  | | Miss. | 12.6% | 55 | 12.2% | | 53 | | 14.0% | 61 |  |  |  |
| **PIP2 – GS score** | | | | | | | | | | | | | |
| **Palmar** | | 0 | 62.1% | 270 | 71.3% | | 310 | | 70.6% | 307 | < 0.001 | 0.058 | 1.000 |
|  | | 1 | 24.8% | 108 | 20.0% | | 87 | | 20.5% | 89 |  |  |  |
|  | | 2 | 6.2% | 27 | 4.4% | | 19 | | 4.8% | 21 |  |  |  |
|  | | 3 | 3.9% | 17 | 1.4% | | 6 | | 1.4% | 6 |  |  |  |
|  | | Miss. | 3.0% | 13 | 3.0% | | 13 | | 2.8% | 12 |  |  |  |
| **PIP2 – PD score** | | | | | | | | | | | | | |
| **Dorsal** | | 0 | 64.1% | 279 | 69.0% | | 300 | | 68.3% | 297 | < 0.001 | 0.943 | 1.000 |
|  | | 1 | 4.8% | 21 | 1.6% | | 7 | | 2.3% | 10 |  |  |  |
|  | | 2 | 0.7% | 3 | 0.2% | | 1 | | - | - |  |  |  |
|  | | 3 | 0.5% | 2 | - | | - | | - | - |  |  |  |
|  | | Miss. | 29.9% | 130 | 29.2% | | 127 | | 29.4% | 128 |  |  |  |
| **Palmar** | | 0 | 73.3% | 319 | 79.5% | | 346 | | 78.4% | 341 | < 0.001 | 0.364 | 1.000 |
|  | | 1 | 7.8% | 34 | 4.6% | | 20 | | 5.5% | 24 |  |  |  |
|  | | 2 | 2.5% | 11 | 0.2% | | 1 | | 0.5% | 2 |  |  |  |
|  | | 3 | 0.5% | 2 | - | | - | | 0.2% | 1 |  |  |  |
|  | | Miss. | 15.9% | 69 | 15.6% | | 68 | | 15.4% | 67 |  |  |  |
| **PIP3 – GS score** | | | | | | | | | | | | | |
| **Palmar** | 0 | | 59.8% | 260 | 69.2% | 301 | | 69.9% | | 304 | < 0.001 | 0.030 | 1.000 |
|  | 1 | | 22.5% | 98 | 19.3% | 84 | | 18.9% | | 82 |  |  |  |
|  | 2 | | 11.0% | 48 | 6.0% | 26 | | 6.0% | | 26 |  |  |  |
|  | 3 | | 3.9% | 17 | 2.3% | 10 | | 2.3% | | 10 |  |  |  |
|  | Miss. | | 2.8% | 12 | - | - | | - | | 13 |  |  |  |
| **PIP3 – PD score** | | | | | | | | | | | | | |
| **Dorsal** | 0 | | 64.4% | 280 | 68.7% | 299 | | 69.4% | | 302 | < 0.001 | 0.799 | 1.000 |
|  | 1 | | 5.1% | 22 | 1.4% | 6 | | 1.6% | | 7 |  |  |  |
|  | 2 | | 1.1% | 5 | 0.7% | 3 | | - | | - |  |  |  |
|  | Miss. | | 29.4% | 128 | 29.2% | 127 | | 29.0% | | 126 |  |  |  |
| **Palmar** | 0 | | 74.5% | 324 | 80.0% | 348 | | 80.2% | | 349 | 0.002 | 1.000 | 1.000 |
|  | 1 | | 8.0% | 35 | 3.7% | 16 | | 3.7% | | 16 |  |  |  |
|  | 2 | | 1.4% | 6 | 0.5% | 2 | | 0.5% | | 2 |  |  |  |
|  | 3 | | 0.0% | 0 | 0.2% | 1 | | 0.2% | | 1 |  |  |  |
|  | Miss. | | 16.1% | 70 | 15.6% | 68 | | 15.4% | | 67 |  |  |  |
| **MTP2 – GS score** | | | | | | | | | | | | | |
| **Dorsal** | 0 | | 48.5% | 211 | 51.0% | 222 | | 53.1% | | 231 | 0.002 | 0.131 | 1.000 |
|  | 1 | | 23.0% | 100 | 27.1% | 118 | | 24.8% | | 108 |  |  |  |
|  | 2 | | 15.4% | 67 | 12.2% | 53 | | 11.7% | | 51 |  |  |  |
|  | 3 | | 9.7% | 42 | 4.8% | 21 | | 6.4% | | 28 |  |  |  |
|  | Miss. | | 3.4% | 15 | 4.8% | 21 | | 3.9% | | 17 |  |  |  |
| **MTP2 – PD score** | | | | | | | | | | | | | |
| **Dorsal** | 0 | | 67.1% | 292 | 74.7% | 325 | | 75.6% | | 329 | < 0.001 | 0.313 | 1.000 |
|  | 1 | | 10.3% | 45 | 5.1% | 22 | | - | | - |  |  |  |
|  | 2 | | 3.2% | 14 | 0.9% | 4 | | - | | - |  |  |  |
|  | 3 | | 0.2% | 1 | 0.2% | 1 | | 0.2% | | 1 |  |  |  |
|  | Miss. | | 19.1% | 83 | 19.1% | 83 | | 18.6% | | 81 |  |  |  |
| **MTP5 – GS score** | | | | | | | | | | | | | |
| **Dorsal** | 0 | | 58.9% | 256 | 63.0% | 274 | | 68.0% | | 296 | < 0.001 | 0.033 | 1.000 |
|  | 1 | | 20.2% | 88 | 22.3% | 97 | | 18.2% | | 79 |  |  |  |
|  | 2 | | 10.6% | 46 | 7.1% | 31 | | 6.7% | | 29 |  |  |  |
|  | 3 | | 6.4% | 28 | 2.8% | 12 | | 2.5% | | 11 |  |  |  |
|  | Miss. | | 3.9% | 17 | 4.8% | 21 | | 4.6% | | 20 |  |  |  |
| **MTP5 – PD score** | | | | | | | | | | | | | |
| **Dorsal** | 0 | | 68.7% | 299 | 74.7% | 325 | | 75.9% | | 330 | < 0.001 | 0.565 | 1.000 |
|  | 1 | | 7.6% | 33 | 3.9% | 17 | | 3.7% | | 16 |  |  |  |
|  | 2 | | 1.8% | 8 | 0.7% | 3 | | 1.6% | | 7 |  |  |  |
|  | 3 | | 1.6% | 7 | 0.5% | 2 | | 0.0% | | 0 |  |  |  |
|  | Miss. | | 20.2% | 88 | 20.2% | 88 | | 18.9% | | 82 |  |  |  |

*GS = greyscale, PD = power Doppler, T0 = baseline, T1 = 3 months visit, T2 = 6 months visit, MCP = metacarpophalangeal, PIP = proximal interphalangeal, MTP = metatarsophalangeal*

**Supplementary Table 3.2 Tenosynovitis/paratenonitis at baseline, 3 and 6 months, change of tenosynovitis/paratenonitis, all (n = 435)**

| **Tendon** | **Grade** | **Baseline (T0)** | | | **3 months (T1)** | | **6 months (T2)** | | | **p-value** | **p-value** | | **p-value** |
| --- | --- | --- | --- | --- | --- | --- | --- | --- | --- | --- | --- | --- | --- |
|  |  | **Prop.** | | **N** | **Prop.** | **N** | **Prop.** | **N** | | **(T0-T2)** | **(T0-T1)** | | **(T1-T2)** |
| **Wrist – GS score** | | | | | | | | | | | | | |
| **EDC** | Yes | 30.1% | | 131 | 18.2% | 79 | 16.6% | 72 | | < 0.001 | < 0.001 | | 1.000 |
|  | No | 65.7% | | 286 | 78.6% | 342 | 80.5% | 350 | |  |  |  |  |
|  | Miss. | 4.1% | | 18 | 3.2% | 14 | 3.0% | 13 | |  |  |  |  |
| **FTS/P** | Yes | 20.9% | | 91 | 10.1% | 44 | 9.9% | 43 | | < 0.001 | < 0.001 | | 1.000 |
|  | No | 75.4% | | 328 | 86.7% | 377 | 86.9% | 378 | |  |  |  |  |
|  | Miss. | 3.7% | | 16 | 3.2% | 14 | 3.2% | 14 | |  |  |  |  |
| **ECU** | Yes | 24.8% | | 108 | 17.9% | 78 | 15.4% | 67 | | < 0.001 | 0.002 | | 0.741 |
|  | No | 71.3% | | 310 | 78.2% | 340 | 81.8% | 356 | |  |  |  |  |
|  | Miss. | 3.9% | | 17 | 3.9% | 17 | 2.8% | 12 | |  |  |  |  |
| **Wrist – PD score** | | | | | | | | | | | | | |
| **EDC** | 0 | 66.7% | | 290 | 73.1% | 318 | 73.8% | 321 | | 0.003 | 0.891 | | 1.000 |
|  | 1 | 6.9% | | 30 | 5.1% | 22 | 4.6% | 20 | |  |  |  |  |
|  | 2 | 3.7% | | 16 | 0.7% | 3 | 0.9% | 4 | |  |  |  |  |
|  | 3 | 0.5% | | 2 | 0.5% | 2 | 0.5% | 2 | |  |  |  |  |
|  | Miss. | 22.3% | | 97 | 20.7% | 90 | 20.2% | 88 | |  |  |  |  |
| **FTS/P** | 0 | 69.7% | | 303 | 74.3% | 323 | 74.0% | 322 | | 0.041 | 1.000 | | 1.000 |
|  | 1 | 5.7% | | 25 | 4.4% | 19 | 4.8% | 21 | |  |  |  |  |
|  | 2 | 1.8% | | 8 | 0.2% | 1 | 0.5% | 2 | |  |  |  |  |
|  | 3 | 0.2% | | 1 | - | - | - | - | |  |  |  |  |
|  | Miss. | 22.5% | | 98 | 21.1% | 92 | 20.7% | 90 | |  |  |  |  |
| **ECU** | 0 | 67.6% | | 294 | 72.4% | 315 | 74.5% | 324 | | 0.001 | 0.853 | | 1.000 |
|  | 1 | 5.5% | | 24 | 5.1% | 22 | 3.9% | 17 | |  |  |  |  |
|  | 2 | 4.1% | | 18 | 0.7% | 3 | 0.9% | 4 | |  |  |  |  |
|  | 3 | 0.9% | | 4 | 0.5% | 2 | - | - | |  |  |  |  |
|  | Miss. | 21.8% | | 95 | 21.4% | 93 | 20.7% | 90 | |  |  |  |  |
| **Finger tendons – GS score** | | | | | | | | | | | | | |
| **ET2** | Yes | 12.6% | | 55 | 6.4% | 28 | 4.1% | 18 | | < 0.001 | < 0.001 | | 0.805 |
|  | No | 83.2% | | 362 | 90.8% | 395 | 92.6% | 403 | |  |  |  |  |
|  | Miss. | 4.1% | | 18 | 2.8% | 12 | 3.2% | 14 | |  |  |  |  |
| **FT2** | Yes | 18.4% | | 80 | 9.7% | 42 | 6.4% | 28 | | < 0.001 | < 0.001 | | 0.333 |
|  | No | 77.5% | | 337 | 87.4% | 380 | 90.3% | 393 | |  |  |  |  |
|  | Miss. | 4.1% | | 18 | 3.0% | 13 | 3.2% | 14 | |  |  |  |  |
| **ET3** | Yes | 10.8% | | 47 | 4.4% | 19 | 3.7% | 16 | | < 0.001 | < 0.001 | | 1.000 |
|  | No | 84.8% | | 369 | 92.9% | 404 | 92.9% | 404 | |  |  |  |  |
|  | Miss. | 4.4% | | 19 | 2.8% | 12 | 3.4% | 15 | |  |  |  |  |
| **FT3** | Yes | 14.7% | | 64 | 7.1% | 31 | 5.3% | 23 | | < 0.001 | < 0.001 | | 0.715 |
|  | No | 79.8% | | 347 | 89.9% | 391 | 91.0% | 396 | |  |  |  |  |
|  | Miss. | 5.5% | | 24 | 3.0% | 13 | 3.7% | 16 | |  |  |  |  |
| **Finger tendons – PD score** | | | | | | | | | | | | | |
| **ET2** | 0 | 66.9% | 291 | | 70.8% | 308 | 73.6% | | 320 | < 0.001 | 1.000 | 1.000 | |
|  | 1 | 3.9% | 17 | | 1.4% | 6 | 0.5% | | 2 |  |  |  |  |
|  | 2 | - | - | | 0.2% | 1 | - | | - |  |  |  |  |
|  | Miss. | 29.2% | 127 | | 27.6% | 120 | 26.0% | | 113 |  |  |  |  |
| **FT2** | 0 | 65.5% | 285 | | 67.8% | 295 | 71.0% | | 309 | 0.390 | - | - | |
|  | 1 | 4.6% | 20 | | 2.1% | 9 | 1.6% | | 7 |  |  |  |  |
|  | 2 | - | - | | 0.5% | 2 | 0.2% | | 1 |  |  |  |  |
|  | 3 | 0.2% | 1 | | 0.2% | 1 | 0.2% | | 1 |  |  |  |  |
|  | Miss. | 29.7% | 129 | | 29.4% | 128 | 26.9% | | 117 |  |  |  |  |
| **ET3** | 0 | 67.4% | 293 | | 70.6% | 307 | 73.1% | | 318 | 0.012 | 1.000 | 1.000 | |
|  | 1 | 2.8% | 12 | | 1.6% | 7 | 0.9% | | 4 |  |  |  |  |
|  | 2 | 0.5% | 2 | | 0.2% | 1 | - | | - |  |  |  |  |
|  | Miss. | 29.4% | 128 | | 27.6% | 120 | 26.0% | | 113 |  |  |  |  |
| **FT3** | 0 | 65.1% | 283 | | 67.8% | 295 | 71.0% | | 309 | 0.015 | 1.000 | 1.000 | |
|  | 1 | 3.7% | 16 | | 1.1% | 5 | 1.1% | | 5 |  |  |  |  |
|  | 2 | 0.2% | 1 | | 0.7% | 3 | - | | - |  |  |  |  |
|  | 3 | 0.5% | 2 | | 0.2% | 1 | - | | - |  |  |  |  |
|  | Miss. | 30.6% | 133 | | 30.1% | 131 | 27.8% | | 121 |  |  |  |  |

*GS = greyscale, PD = power Doppler, T0 = baseline, T1 = 3 months visit, T2 = 6 months visit, FT/ET2 = flexor/extensor tendon on MCP2 level, FT/ET3 = flexor/extensor tendon on MCP3 level, FDS/P = flexor digitorum superficialis/profundus tendon, EDC = extensor digitorum communis tendon (extensor compartment lV), ECU = extensor carpi ulnaris tendon (extensor compartment Vl)*

**Supplementary Table 4: SRM with 95% Cl after 3 months, 3 to 6 and 6 months**

| **mode** | **Combination** | **n** | **SRM (95% CI)**  **Baseline to 3 months**  **(T0 -T1)** | **n** | **SRM (95% CI)**  **3 to 6 months**  **(T1-T2)** | **n** | **SRM (95% CI)**  **Baseline to 6 months**  **(T0-T2)** |
| --- | --- | --- | --- | --- | --- | --- | --- |
| **GS** | GS1 | 407 | 0,330 (0,232 – 0,425) | 409 | 0,122 (0,023 – 0,218) | 409 | 0,377 (0,279 – 0,472) |
|  | **GS2** | **392** | **0,395 (0,297 – 0,497)** | **398** | **0,129 (0,033 – 0,288)** | **397** | **0,433 (0,335 – 0,530)** |
|  | GS3 | 404 | 0,346 (0,249 – 0,443) | 404 | 0,129 (0,032 – 0,222) | 407 | 0,385 (0,285 – 0,484) |
|  | GS4 | 389 | 0,395 (0,294 – 0,496) | 393 | 0,132 (0,035 – 0,233) | 395 | 0,433 (0,332 – 0,530) |
|  | GS5 | 377 | 0,413 (0,315 – 0,511) | 389 | 0,152 (0,051 – 0,252) | 381 | 0,456 (0,354 – 0,557) |
| **PD** | PD1 | 355 | 0,317 (0,213 – 0,424) | 353 | 0,116 (0,012– 0,221) | 350 | 0,406 (0,305 – 0,507) |
|  | PD2 | 299 | 0,349 (0,233 – 0,462) | 304 | 0,127 (0,016 – 0,234) | 298 | 0,420 (0,306 – 0,538) |
|  | PD3 | 293 | 0,321 (0,209 – 0,434) | 296 | 0,123 (0,008 – 0,236) | 287 | 0,424 (0,308 – 0,534) |
|  | PD4 | 351 | 0,309 (0,205 – 0,417) | 349 | 0,161 (0,052 – 0,267) | 346 | 0,447 (0,343 – 0,550) |
|  | PD5 | 296 | 0,328 (0,219 – 0,442) | 300 | 0,178 (0,062 – 0,289) | 285 | 0,445 (0,334 – 0,559) |
|  | PD6 | 289 | 0,310 (0,194 – 0,425) | 293 | 0,202 (0,091 – 0,318) | 284 | 0,462 (0,344 – 0,584) |
|  | PD7 | 256 | 0,345 (0,222 – 0,470) | 265 | 0,202 (0,082 – 0,321) | 258 | 0,480 (0,355 – 0,603) |
|  | PD8 | 213 | 0,263 (0,129 – 0,400) | 218 | 0,210 (0,076 – 0,342) | 210 | 0,414 (0,282 – 0,553) |
|  | **PD9** | **259** | **0,363 (0,242 – 0,487)** | **268** | **0,126 (0,005 – 0,240)** | **260** | **0,442 (0,324 – 0,564)** |
| **DAS28** |  | 394 | 0,728 (0,630 – 0,826) | 383 | 0,102 (0,002 – 0,202) | 395 | 0,731 (0,632 – 0,830) |

*GS = greyscale, PD = power Doppler, SRM = standardized response mean, T0 -T1 = baseline to 3 months 3 visit, T1 -T2 = 3 months to 6 months-visit, T0-T2 = baseline to 6 months visit, combinations in bold were included in the reduced score*
